# Self-Care Practices among Diabetes Patients in Addis Ababa: A Qualitative Study

**DOI:** 10.1371/journal.pone.0169062

**Published:** 2017-01-03

**Authors:** Dagmawit Tewahido, Yemane Berhane

**Affiliations:** Addis Continental Institute of Public Health, Addis Ababa, Ethiopia; Weill Cornell Medical College Qatar, QATAR

## Abstract

**Background:**

Self-care practices that include self-monitoring of blood sugar level, diet management, physical exercise, adherence to medications, and foot care are the cornerstones of diabetes management. However, very little is known about self-care in developing countries where the prevalence of diabetes is increasing.

**Objective:**

The objective of this study was to describe self-care practices among individuals with type II diabetes in Addis Ababa, Ethiopia.

**Methods:**

A qualitative method was used to gather data from type II diabetes patients. Patients were recruited from the outpatient diabetes clinics of two public hospitals in Addis Ababa. Data were collected using a semi structured interview guide. A thematic analysis approach was used to process the data.

**Results:**

Overall self-care practices were not adequate. Most patients reported irregular self-monitoring of blood sugar. Dietary and physical exercise recommendations were inadequately practiced by most of the participants. Most patients better adhered to medication prescriptions. Patients generally lack proper information/knowledge regarding the importance of self-care and how it should be implemented. Based on reported behavior we identified three main categories of patients; which are those ‘endeavor to be compliant’, ‘confused’ and ‘negligent’.

**Conclusion:**

Diabetes patients largely depend on prescribed medications to control their blood sugar level. The importance of proper self-care practices for effective management of diabetes is not adequately emphasized in diabetes care centers and patients lack sufficient knowledge for proper self-care.

## Introduction

Globally over 14 million people die each year from non-communicable diseases such as diabetes mellitus between the ages of 30 and 70, of which 85 per cent are in developing countries [1]. Ethiopia is among the top five countries with the highest number of people affected by diabetes mellitus in Sub Saharan Africa [2]. As a result hospital admissions for diabetes management has been rising in recent years [3, 4].

Effective management of diabetes requires strong and consistent cooperation of the patient [5]. Often the complications associated with diabetes management are highly attributable to the failure to comply with self-care recommendations [6]. The poor self-discipline, and lack of support from family members and/or physicians, poverty and lack of access to health facility are some of the major for failure to comply [7].

There are well recognized and specific self-care components to prevent and/or delay complications and possibility of early death associated with diabetes. The components are self-monitoring of blood glucose level, diet control, optimum physical exercise, adherence to medication/s and proper foot care [8]. Various strategies were adopted in different countries to help people with diabetes improve their self-care practices depending on the context [9]. Implementation of a comprehensive patient education program was reported to have enhanced diabetes self-care practices [10]. Improved social support for patients with diabetes has facilitated diabetes self-care and achieve improved glycemic control [11]. Task shifting is another approach successfully implemented to improve self-care in places where doctors have heavy work load; either nurses or other health professionals were specifically trained to provide proper information to the patient instead of the busy doctor [12]. Understanding patients self-care cultural and value systems is another important factor to designing a responsive program that can influence their diet and exercise choices, trend of blood glucose monitoring, and compliance with prescribed medication regimens [9]. While the burden of diabetes is increasing in Ethiopia, studies conducted to understand self-care practices are very limited. Thus the aim of this study was to describe the diabetes self-care practices and identify facilitators and barriers to the practice among type II diabetes patients attending follow-up in public hospitals.

## Methods

The study was conducted in Addis Ababa, the capital city of Ethiopia. There were five public hospitals that ran a special diabetes follow up clinics at the time of the study where patients are appointed every four to six months to see their doctor and receive services in outpatient departments. The clinics provide diabetes education on self-care practices and on proper self-injection techniques. We selected Menelik II and Zewditu memorial hospitals for this study. These two hospitals have been running a separate diabetes follow up clinics for more than three decades. In Ethiopia all public hospitals provide consultation and anti-diabetes medications free of charge. However additional diagnostic treatment services may not necessarily be free unless the patient has a ‘certificate of poverty’ from local administration.

The study participants were patients with type II diabetes that came to follow up clinics between November 2013 and February 2014. The inclusion criteria were having been diagnosed with type II diabetes for at least five years and being between the ages of 35 and 65 years. Patients who were not in a good physical/mental condition during the study time were excluded. As the aim of the study was to describe the day to day self-care practices of patients with diabetes, newly diagnosed patients were not included in the study.

A purposive sampling procedure was used to recruit patients for the study based on their age, sex, educational level, income level, duration of diagnosis/follow up and patient’s personal behaviors. This was done to be able to see the different forms of self-care practices from different perspectives. The doctors and nurses working in the follow up clinics helped in identifying patients that fulfil the inclusion criteria of the study. We tried to include patients believed to be interactive, open minded and those who were willing to participate in the study. Each person identified as potential respondent was then individually asked for consent after being informed about the purpose and the required procedures.

The semi-structured interview guide used for data collection was developed by reviewing relevant literatures. In addition, participants were encouraged to speak up their mind in case they had anything further to tell related to the topic. A pilot was conducted in another public hospital to assess the acceptability and ease of understanding the interview process. Then interview guide was revisited to make the necessary changes and modifications before conducting the actual interviews included in the analysis.

All interviews were conducted by the first author in a private space within the hospital compound. The interviews were conducted in Amharic language, which is the national language of Ethiopia. All interviews were tape recorded with verbal consent obtained from the study participants. In addition relevant notes were taken during the interview to document key issues and observations. Interviews took approximately 45–60 minutes. Interviews were conducted until the point of relative saturation with regard to the issues being discussed.

All interviews were transcribed verbatim in Amharic and then translated into English for data analysis. The translated content was coded manually and entered into a computer software (open-code) used for qualitative data sorting. Codes were given and grouped into categories that were predefined based on the objective of the study. The field notes were included in the memos section of the software. Coding started by a thorough reading of each interview material, followed by line-by-line flagging of each of the interviews. A coding procedure was established jointly by the co-authors.

A thematic analysis approach was used to categorize the codes thorough several iterations. The thematic areas were diet, physical exercise, medication adherence, self- monitoring of blood glucose and foot care. Patients were identified and labeled as “Negligent”, “Confused” and “Endeavour to be compliant” based on their personal coping methods with respect to their self-care practices that emerged during the analysis process.

Ethical clearance for the study was obtained from Institutional Review Board of Addis Continental Institute of Public Health and permission to conduct the study was granted by the Addis Ababa city administration Health Bureau. Interviews were conducted after participants provided verbal informed consent. The interviews were conducted in manners that assured privacy for the respondents. Access to raw data was restricted only to the study investigators.

## Results

A total of thirteen in-depth interviews were conducted with type II diabetes patients. All participants have had diabetes for at least five years. Seven of the participants were female. All respondents were between the age of 35and 65, and nine of them were married. With regard to their educational back ground, one has attended college, six had elementary to high school education, and three could not read and write. Participants were from various religious and ethnic groups. None of the patients invited for the study refused to participate in the study.

### Self-monitoring of blood sugar

Participants generally reported that they do not regularly check their blood glucose level. Even patients who had their own gluco-meter machine at home reported testing their blood sugar once every 4–6 six weeks. Those who do not own the glucometer machine at home reportedly go to either a nearby private clinic or laboratory only when they feel ill. About a third of the study participants reported checking their blood sugar level only during their follow up visits to the hospital, which is every three to four months. These findings indicate blood sugar monitoring is irregular and the high risks to develop long term diabetes complication due to poor glycemic control.

*“If my sugar reads above 250 when I get it tested*, *I adjust my dose; take a little bit more of my insulin since the current dose is obviously not enough for me*. *And when it gets down*, *I lower it back to the previous dose*. *They tell you not to do that but I have been monitoring my sugar like that for all these years*.*”* 58-year-oldf self-monitoring male participant

Another patient described her experience of self-monitoring of her blood sugar as frustrating. She felt that controlling her blood sugar was beyond their capability.

*“My blood sugar fluctuates a great deal*. *No matter what I do to control it*, *nothing prevents it from shooting up high*. *It’s out of my control*. *So I’ve left it for the doctors to take care of it*. *All I can do is take the prescribed medications*.*”* 44-year-old female respondent

### Dietary practices

Most study participants recognized diet as an essential component of self-care practice for people with diabetes. Almost all respondents reported to have totally avoided taking table sugar and minimized intake of sweet drinks and food. Almost all participants described their usual diet to consist of *Injera* (a staple food in most parts of Ethiopia which is made of a locally grown grain *‘teff’*) every day of the week. A diabetes friendly meal plans were not widely recognized by most patients. Only one participant had an idea of the approximate amount of proteins, carbohydrates and fats recommended for people with diabetes. None of the participants reported any kind of system/meal plan that considers their diabetes. The following are illustrative quotes on dietary practices:

*“…I don’t like stressing too much over what I eat*. *Like I told you*, *as long as you avoid sugary foods and drinks…”* 50-year-old male participant

“Sugar for a ‘sugar’ (diabetic) patient? That is like facilitating your journey to your grave…

*Everybody here knows that sugar is absolutely to be not touched …”* 40-year-old female participant

*“Starchy foods are not allowed for us* (patients with diabetes)*…I try to be careful with food items such as potatoes*, *wheat and corn*.*”* 56-year-old female respondent

Lack of awareness/information was the most common reason mentioned for not following a diabetes friendly diet. Other reasons include inconveniences at workplaces, personal food preferences, family meal preparation habits, low income, negligence, and temptations.

*“Up to now*, *I have not retreated from what I like to eat*. *I eat like everybody else*. *I eat what I like and don’t want to be picky saying ‘I am diabetic patient’ every time I sit for a meal*.*”* 45-year-old female respondent

The pressure during social gatherings was a concern to some study participants. Sharing food during social gathering in Ethiopia is considered a way of expressing respect and affection to one another and refusing to eat from a common dish is 'unacceptable'.

*“I can’t go to a ‘mahber’* (social event) *for instance and say ‘I won’t eat or drink’*. *I take what they give me with pleasure because it is not appropriate to refuse*, *as the saying goes*, *‘yeweledutin kalsamulet*, *yakerebutin kalbelulet’*, (a guest is disrespectful… if failed kissing the host’s children or if refused eating food served by the host) *therefore I go and I eat what they have prepared*. *A social life is essential for us*.” 56-year-old female respondent

Preparing separate meals for one person in a family is a practical challenge. In addition as the Ethiopian culture does not encourage men to participate in food preparation or to be seen in the kitchen. That means a man with diabetes has to eat whatever is served to the family.

*“I eat what my husband and children eat*. *I cannot prepare a separate meal just for myself; you know it just is not convenient*.*”* 45-year-old female participant

Some of the participants also reported that adhering strictly to diabetes dietary recommendations is boring and practically impossible; food restrictions intensify their cravings and make life more stressful.

*“I am not going to lie to you*, *I like alcohol*. *The doctor always warns me that ‘arekie’* (a local Gin) *is worsening my illness*, *and I know how my sugar increases after drinking*. *But I am fed up of living everyday thinking about my illness*. *So when I get bored I say to myself ‘If I am going to die anyway*, *why not enjoy life a bit’ and I go out to drink*.*”* 52-year-old male participant

### Practices with regard to regular physical exercise

Nearly all informants admitted that they do not exercise regularly. The most commonly mentioned reasons for not doing regular physical exercise were lack of interest, lack of motivation, busy work schedule, not being able to afford gymnasium fees and not convinced that exercise is important.

*“I know that exercise is necessary*. *I have also been told to exercise since I also have high blood pressure*. *I have started many times to regularly exercise*, *but it doesn’t last*. *I get tired of it fast*, *or something comes up to force me stop*. *There after it is just too discouraging to start all over again*.*”* 56-year-old female participant

*“I don’t exercise regularly*. *I take occasional walks when I have the time but serious sport is not in my schedule*.*”* 52-year-old male respondent

*“Even if I was committed to regular exercise*, *it is not convenient*. *There is no place to exercise in the city and the gyms are not affordable*.*”* 44-year-old male respondent

*“Exercise at my age?! What difference would it make after all these years*, *unless I want to break my old bones*?!…*”* 58-year-old male participant

### Taking diabetes medication regularly

Most of the respondents consider their anti-diabetes medications as the most vital element of the diabetes management and their survival. The majority reported they are complying most with instructions regarding medicines more than any of the other components of self-care practices.

*“I never omit my medication on purpose*. *I know going on and off on diabetic medications put your life in dangerous situations like accidental fainting or even death*.*”* 56-year-old female participant

The participant stated erratic use of medications and adjusting doses by themselves, is a common occurrence to make up for their unhealthy dietary practices and to correct blood sugar levels.

*“I mostly follow the doctor’s orders*. *But when it (blood sugar) is unacceptably high*, *let’s say above 250*, *then I slightly increase the dose*.*”* 58-year-old male participant

Most participants who were on insulin also mentioned missing their doses when traveling away from home due to lack of cold storage/refrigerator.

*“When I go out for a field work* (away from home) *for 2 or 3 days*, *I may not have my insulin with me*. *It’s not comfortable to go around with injection equipment*. *Besides there isn’t refrigerator and stuff…for keeping the medication”* 38-year-old male participant

Another common challenge mentioned by many participants was the injection site pain and abscess resulting from the daily insulin injections. Patients were frustrated and scared when the pain became too much and especially when they faced visible signs such as swelling and abscess, at the injection sites.

*“…not faced abscess so far but the pain is unspeakable*. *And pricking yourself like a piece of clothing all your life is not something enjoyable*. *My thighs and abdomen bruise from time to time*.*”* 52-year-old male participant

*“…a couple of years ago my thigh got irritated and had pus*. *It was terrible*. *It took it a while to heal but I never forgot the pain*, *and what kills you more is the fear of that pus never drying and costing you your leg…”* 60-year-old female participant

More than half of the study participants reported taking additional medication to control/treat other related conditions such as hypertension, high cholesterol and other heart conditions, which they mentioned could be barriers to adhering to their anti-diabetes medications. The patients widely mentioned cost and availability of medications as a serious challenge in addition to pain and abscess at injections sites.

*“…The drugs are not affordable…besides diabetes doesn’t come alone*. *There’s the cholesterol and hypertension that come along*. *I struggle to cover all that with my government salary*.*”* 52-year-old male participant

### Regular foot care

Foot care was the least recognized self-care practice by the study participants. Most have not even heard of what foot care is, although many of them have reported foot injury as one of the common health problems for them.

*“I know I have to be careful of sharp things and ‘enqfat’* (street hurdle) *when I walk in the street since injury to my foot can eventually develop into gangrene*. *But it is impossible to always move flawless…”* 56-year-old female participant

Female study participants more than male study participants reported to have been caring about foot hygiene and give more attention to choosing appropriate footwear.

*“Most evenings after I am done with my house work*, *I wash my feet and dry them*. *I watch for any ‘chok’* (fungus) *between my fingers*. *I use nail clippers to cut my nails regularly…”* 45-year-old female respondent

A few participants had experience of some bad foot wound; one of them had to have leg amputation due to severe complication. Study participants said foot ulcers were inevitable to a person with diabetes sooner or later.

*“…if you are unfortunate like me*, *a food ulcer can lead to your leg cut off”* 50-year-old male participant

### Participants experiences of the self-care practices

We observed that study participants behaved in different ways in coping with their illness and diabetes self-care. We categorized them into three different groups based on their utterances as: ‘Negligent’, ‘Confused’ and ‘Endeavour to be compliant’. This grouping helps to see their relative level of self-care in relation to their illness coping strategies, as well as their attitude towards self-care (Table 1).

**Table 1:** category by utterances and respective characteristics.

Utterances Category	Characteristics
Negligent	• Disinterested, not motivated to know about self-care practices • Do not take actions, put blame on God or fate • Mostly ignore self-care practices • Refuse to discuss with peers or join diabetes association
Confused	• Anxious/fearful about complications • Do not know what it is and how to do self-care prctices • Minimal self-care practices • Know there is diabetes association but are not involved
Endeavor to be compliant	• Discuss with peers/doctors/participate in diabetes association activities to know more about self-care practices • Better informed and aware of their responsibilities for self-care • Moderate self-care practices

## Discussion

Overall, a comprehensive self-care practice among diabetes patients was uncommon. Most of the respondents entirely depended on their medications to manage their illnesses and tend to undermine the importance of the other elements of self-care either due to lack of resource/poverty, lack of awareness, lack of support or negligence [5].

The irregularity of blood sugar monitoring was the main shortcoming of diabetes control in this study. This is a precursor to the development of long term diabetes complications of diabetes. As reported elsewhere in sub Saharan Africa and in Ethiopia, irregular blood sugar measurement was related with the lack of personal glucometers or lack of easy access to health facilities and laboratories [8]. Long intervals between clinic appointments was also reported as one of the reasons for taking the responsibility of self-adjusting medication dosages by patients with diabetes. Provision of a comprehensive education program and task shifting from physicians to nurses or to a person specifically trained to perform a limited task such as delivery of diabetes education was found to be helpful improving patients care in busy diabetes clinics in Sub-Saharan African countries [13].

Food habits in the family and personal food preferences were among the serious challenges which made dietary adjustment difficult for people with diabetes. Participation in social gatherings and food related socio-cultural norms could pose serious impediments to effective diabetic control in Sub-Saharan Africa [9, 12]. In addition shortage of cash to purchase food items appropriate for persons with diabetes, craving for cultural/traditional food and limited availability of variety of food items in the local market are barriers to dietary self-care practices as seen from this as well as other studies in similar settings [12].

Physical exercise, regardless of weight or body mass index, is critical to effectively control blood sugar level and in reducing persistent hyperglycemia [2, 12]. Lack of appropriate information and lack of motivation to engage in a regular physical exercise are common short comings of diabetes self-care practices [14]. Although most people in Africa walk on foot every day, illness and old age can eventually significantly reduce patients’ ability to walk as usual [12]. For aged and ill individuals going to a gym regularly may not be feasible due to either cost or physical distance. Thus, appropriate guidance needs to be given for the kind of exercise that can be done at home [15].

Injection site pain and abscess are common side effects that impede strict medication adherence among people with diabetes [16]. However, adherence to anti-diabetes medication was better of all self-care practices [17]. This could be due to either over reliance on medication or its free availability, or the ease to practice it compared to the other components which require more commitment [18].

Foot care was the least practiced diabetes self-care in our setting. This could be due to lack of proper understanding of its importance or the consequences by persons with diabetes [18]. In Sub-Saharan Africa foot wound gets often complicated resulting in severe infection/sepsis gangrene [2]. A proper diabetes education has shown a promising improvement on foot self-care practice [19].

Studies show that persons with diabetes experience disproportionately high rates of social and emotional difficulties compared to the general population. Negative emotions such as frustration and feeling of helplessness contributed to poor self-care practices including poor blood sugar monitoring [7, 20].

We grouped the behavior of persons with diabetes in to three based on their utterances: The ‘Endeavor to be compliant’ reach out to other people and diabetes association to discuss their condition and could build their motivation for positive self-care practices [21].

Those grouped under ‘confused’ were mostly the ones who seemed to be lacking information, although they keenly wanted it. Even if some knew about diabetes association, they were not acquainted with any benefit that they could individually get from them. This lack of information is commonly observed in both developed and developing countries [11]. These patients are tangled in fear and confusion that their self-care practices are not sufficient to assist their diabetes control. This group are likely to be very frightened of the perceived complications [18].

The third group, ‘Negligent’, appeared to resist self-care recommendations; consequently their self-care practice is very limited and uncommitted. These patients ignore their condition (diabetes), and as a result refuse to discuss about it either with peers or join diabetes association. Glycemic control in such patients tend to be poor and their chance of developing complications early is high [18].

In conclusion, Diabetes self-care is generally poor mainly due to insufficient guidance and support provided to persons with diabetes. Greater attention needs to be given to improve patient education and support in diabetes clinics to ensure better self-care practices and avoid early development of complications.

### Strengths of the study

The study took a fresh approach of self-care practices by choosing a qualitative methods from patents’ perspective in their own words; thereby addressing previously unseen sides. There was minimum recall bias due to chronic nature of disease. Diabetes self-care is a relatively non-sensitive issue for patients to freely discuss about, and what’s more, patients were interested and eager to converse about their conditions which facilitated in generating rich data.

### Limitations of the study

Absence of multiple data collection methods, which is limited to interviews to patients enrolled only from public hospitals was a limitation of this study. In addition, social desirability bias may be introduced despite the cautions taken during the interviews.
